# Supplementary material for: CRISPR-finder: A high throughput and cost-effective method to identify successfully edited Arabidopsis thaliana individuals
Source: Quant Plant Biol. 2021 Jan 5;2:e1. doi: 10.1017/qpb.2020.6 (PMC10095899; doi:10.1017/qpb.2020.6)
Supplement: Supplementary file 1 [file qpbsup.zip › S2632882820000065sup001.docx]

**Supplementary Figures**

**
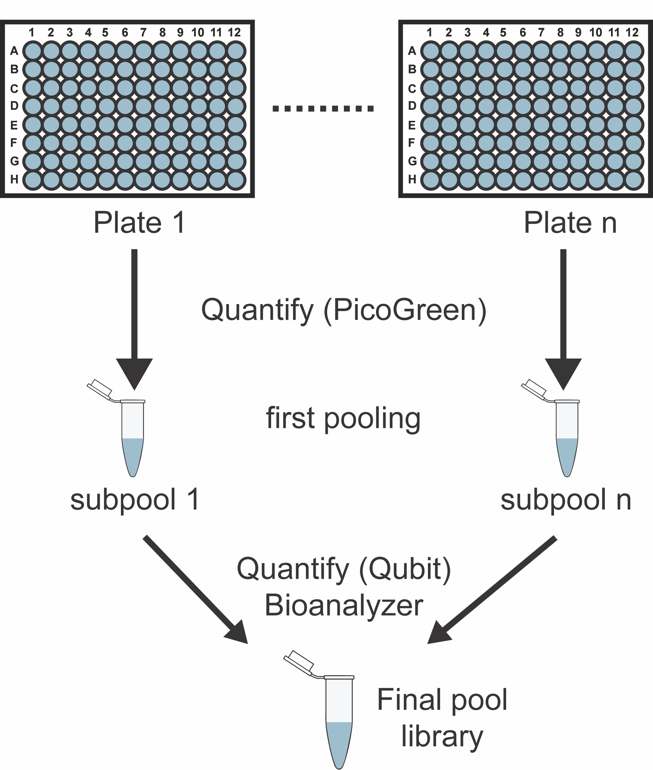
**

**Supplementary Figure 1: Pooling procedure of the separate amplicons.** All the individual amplicons are quantified using PicoGreen. The amplicons of each plate are pooled together for generating the subpools. These subpools are then quantified using Qubit and Bioanalyzer and they are combined to generate the final pooled library. For more information see Materials and Methods.


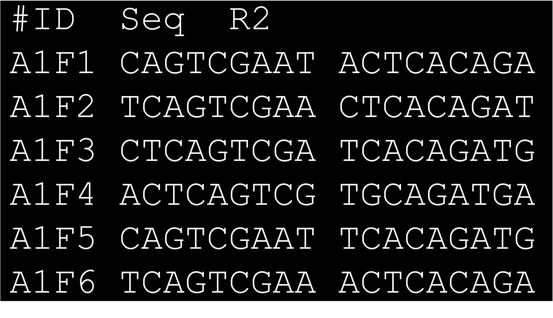


**Supplementary Figure S2: Text file for PlexSeq.** Example of text file that is needed for PlexSeq to demultiplex the raw reads based on the frame-shifting nucleotides. The data consist of three columns. The first column indicates the name of the output file after the demultiplexing. Columns two and three include the first 9 expected nucleotides for each read on the oligonucleotides that were used for the amplification during library preparation.


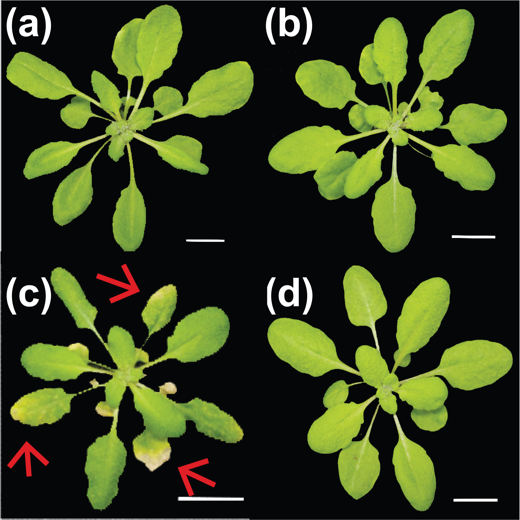


**Supplementary Figure S3: Morphology of *ics1/sid2* mutants.** The plants were growing in 23^o^C SD conditions (8 h light/ 16 h dark). The pictures were taken 40 days after sowing. Chlorotic and necrotic regions on leaves are indicated by red arrows. **(a)** Col-0 reference wild type, **(b)** Col-0 *sid2*-2 mutant, **(c)** TüWa1-2 wild type, and **(d)** TüWa1-2 *ics1*-1c mutant. The scale bars indicate 1 cm; note the different sizes of scale bars.
